# Substance use recovery needs among college students seeking recovery services: a thematic qualitative analysis

**DOI:** 10.1186/s13722-024-00518-x

**Published:** 2025-01-07

**Authors:** Lindsey M. Nichols, Tiffany B. Brown, Angela Allmendinger, Emily A. Hennessy, Emily E. Tanner-Smith

**Affiliations:** 1 Department of Counseling Psychology and Human Services, University of Oregon, Eugene, OR USA; 2 Recovery Research Institute, Center for Addiction Medicine, Department of Psychiatry, Massachusetts General Hospital, Boston, MA USA; 3 Department of Psychiatry, Harvard Medical School, Boston, MA USA; 4 Prevention Science Institute, University of Oregon, Eugene, OR USA

**Keywords:** Collegiate recovery programs, College students, Mental and behavioral health, Substance use recovery, Thematic analysis

## Abstract

**Background:**

College students who are in recovery from substance use disorders face challenges related to abstaining from substance use, finding supportive social networks, and achieving their academic goals. These students may therefore seek out various recovery supports at their institutions to meet their needs and goals.

**Methods:**

This study analyzed previously collected data to explore themes related to students’ experiences of recovery, including their recovery needs and challenges while also attending college. We conducted qualitative thematic analysis of written responses to open-ended prompts posed to 92 college students from one university (47% female; *M* age = 21.5 years, *SD* = 5.6) who participated in a larger parent study of Collegiate Recovery Programs in the United States. We used a phenomenological approach to guide the current study, to characterize the meaning and experience within the shared phenomenon of recovery processes among college students.

**Results:**

Two broad categories emerged, representing nine total themes that were coded: (a) intrapersonal factors: recovery-specific challenges, self-care and coping, mental and behavioral health, life challenges, and personal motivations and attributes; and (b) interpersonal/social factors: 12-step recovery supports, external supports and community, college environment, and relationships with others.

**Conclusions:**

Findings offer insight into barriers and facilitators to recovery among colleges students and are discussed in terms of their implications for primary stakeholders at institutions of higher education to support college students in substance use recovery.

## Background

Problematic substance use and substance use disorders (SUDs) are prevalent among young adults in the United States. In 2021, substance use rates were highest among U.S. young adults ages 18–25 compared to any other age group (e.g., [1]). Specifically, 38% of 18–25-year-olds (12.7 million people) reported illicit drug use (including cannabis) and 67.1% (22.4 million people) reported alcohol use in the past year in 2021. Rates of SUDs in the past year are also highest among this age group: at an estimated 25.6% or 8.55 million U.S. young adults [1]. For those attending institutions of higher education, the college environment is often characterized by heightened levels of social acceptability of alcohol and drugs, and riskier levels of use such as high-intensity drinking [1, 2]. Further, 24% of college students who reported drinking in the past 12 months also reported experiencing two or more consequences when drinking (e.g., blacked out, had unprotected sex, physically injured self; [2]).

In 2021, approximately 2% of U.S. college students indicated being in recovery from substance use [2]. Recovery from substance use is defined here as “a process of change through which people improve their health and wellness, live self-directed lives, and strive to reach their full potential” [3]. For students who identify as being in recovery, the college environment can be particularly difficult to navigate because they must navigate the social milieu, academic demands, and their personal well-being in addition to their recovery goals. Unfortunately, college environments have been considered “abstinence hostile” given cultural norms that normalize pervasive substance use [4]. Indeed, among those students identifying as being in recovery, 51% said they drank alcohol within the last 2 weeks and 17% said they used cannabis within the last 2 weeks [2]. Thus, the college environment itself may represent a barrier for some students in their efforts to abstain from using drugs and/or alcohol.

Collegiate Recovery Programs (CRPs) are one type of program that can offer support for college students in recovery from substance use in this challenging environment. CRPs aim to ameliorate the burden on students struggling with substance use issues while navigating life transitions associated with college and emerging adulthood. With increasing recognition among college health professionals that many students arrive on campus with substance use histories that warrant recovery-oriented, supportive, continuing care approaches [5], CRPs may be well positioned to provide such support. Descriptive and observational research suggests that CRPs tend to reach the most at-risk students [6–8] and that CRP participation is associated with beneficial changes among participants, including increased college retention, decreased substance use, and increased grade point averages [9–12]. For a more detailed description of CRPs, see White and Finch [13], Smock et al. [14], Cleveland et al. [6], and Vest et al. [15].

One guiding framework to conceptualize the supports, needs, and barriers for college students in recovery is recovery capital, which includes internal and external resources that individuals draw upon throughout their recovery journeys [16, 17]. Recovery capital includes personal and interpersonal domains relevant to recovery, as well as community-level factors [18]. To inform what services are offered by CRPs and other mental health support units at institutions of higher education, it is important to understand both the substance use recovery needs and challenges reported by the students who seek such services. This includes an understanding of the characteristics of these students, including co-occurring health conditions, identities, and current supports to provide tailored services. Recent research indicates that students participating in CRPs report high rates of historical and/or current mental health problems [7, 8, 19, 20], and some students report participating in CRPs due to other behavioral health concerns (e.g., disordered eating behaviors, [8, 20] that are negatively associated with academic outcomes (e.g., failed or missed classes, lower grades).

Prior qualitative studies have found that the primary challenges faced by students in recovery are the environmental influences to reengage in substance use and experiences of social isolation and social stigma in college [9, 21], addiction stigma [22], seeking peer support and sense of community [9, 23–26], and balancing recovery and academic priorities [9, 22]. Past research also indicates the salient influence of family dynamics and familial substance use patterns on students in recovery [27–29], as well as general life challenges that add to these recovery challenges [19, 24, 30, 31]. In line with recovery capital, themes have been identified regarding students’ self-efficacy and identify formation while in college and in recovery, as well as personal coping strategies like establishing boundaries [32]. In terms of resources, students have reported that CRPs addressed some of these challenges by providing academic support and a sense of community provided by connections to external sober supports, relational and general academic support from program staff, and on-campus 12-step meetings [9, 32]. Additional resources provided by CRPs include opportunities to build peer social support and recovery-specific peer networks, which can combat stigma or the social expectation to engage in substance use while in college [9, 21, 22, 28].

The existing qualitative literature base regarding CRPs is informative yet nascent, suggesting the need for further investigation into the resources and supports that CRPs could potentially provide to students to improve their academic and recovery outcomes [15]. What is currently absent from the evidence base are robust qualitative studies documenting the unique needs of college students in recovery from larger samples of students who are actively seeking recovery supports. The majority of published qualitative research has utilized data gained from semi-structured interviews from a small sample of students (generally ranging from 4 to 15 participants; [9, 19, 21, 22, 31–33]). While these interviews offer rich findings documenting within-in person experiences, analyzing written response data from a large sample of students in recovery would allow for a deeper analysis of between-person experiences that could inform themes among students in recovery. Analysis of written data has been widely under-utilized in qualitative research yet can offer focused and self-reflective participant information [34]. The ability to analyze and summarize students’ perspectives about what resources these students would find most useful for their academic and recovery journeys would add to the growing qualitative evidence base, and inform future research syntheses, as well practices and policies for students in recovery in institutions of higher education. The present study provides a unique perspective by harnessing data from a large sample of students in college and in recovery who were seeking to join a CRP but had not yet started.

### Aims

The current study provides an analysis of a large number of students’ written responses to questions on their CRP membership applications. These questions were used by the CRP to gauge students’ reasons for seeking recovery, successes/challenges throughout their recovery journey, and to identify challenges of being in recovery and in college to help guide the CRP staff in supporting their members. Utilizing these previously collected qualitative data, the aims of the present study were to explore the experience of recovery from students’ responses to questions about recovery needs, challenges, and experiences while also attending college.

## Methods

### Site recruitment and participants

This study used secondary analysis of data from one CRP derived from a larger parent study (see [12]). In the parent study, we approached 13 CRPs operating in different regions of the United States and invited those sites to participate by sharing de-identified existing data (i.e., data collected as part of their program operations). Of the 13 CRPs approached, six (46%) agreed to participate, two were not interested in participating, and five were interested in participating but did not have authority to share existing data with external collaborators. Only one site collected qualitative data from students via open-ended question responses, and this is the site that contributed data for the analytic sample in the current study. For more information about site recruitment and CRP characteristics from the parent study, see Hennessy et al. [12].

Participants in the current study included college students attending a medium sized 4-year university in the Northeastern United States who were seeking recovery services and applied to the CRP at the university but had not yet been admitted into the CRP and were not participating in any CRP programming. The CRP membership requirements at the university included participation in at least one of the programmatic activities per week, commitment to abstinence-based recovery, and engagement in an active program of recovery. Sober housing was also available at this institution for CRP members.

Data used in the present study were collected between 2011 and 2018. To be eligible for inclusion in the present qualitative study, participants needed to have completed at least one of the five open-ended prompts posed as part of the CRP membership application (outlined below). Of 163 total students for whom data were provided by the site, qualitative data were available for 92 students (56% of total sample).^1^

### Procedure

Because the study included secondary analysis of existing, de-identified data, the Institutional Review Board at the University of Oregon determined that the study did not qualify as human subjects research, and thus no human subjects approval was required.

### Measures

Prospective CRP members completed an application that asked for their age and gender and a set of open-ended questions. Students were posed the following five^2^ open-ended prompts in written format as part of this application: (1) *What led you to seek recovery?* (2) *Describe success and challenges in your recovery journey.* (3) *Describe your personal recovery program.* (4) *What have been the most sustaining elements of your recovery?* (5) *What challenges will you face while trying to be successful in college and maintaining a healthy recovery program? What would be helpful to you in addressing these challenges?*

### Qualitative methodology and analysis

Given our aim to understand the experience of CRP students, the research design and overall methodology was organized using phenomenology [35, 36]. The phenomenon of interest was the meaning and experience of the recovery process of students enrolled in a 4-year university.

Data analysis procedures followed the main steps outlined by Moustakas [36]: (a) each transcription was separated into units of meaning (horizontalization), (b) units were then transformed into statements of meanings, and (c) statements were described as *what* was experienced (textural) and *how* it was experienced (structural). The analysis team (first and second authors) reviewed all open-ended responses for coding so that two researchers coded each transcript. Initially, each coder read transcripts one time and then began the coding process on the second read. After the first round of review, five broad codes were identified (seek recovery, success and challenges with recovery, personal recovery program, sustaining elements of recovery, and success/challenge with college) with 74 subcodes. Initially, the analysis team used open coding methods to independently note the specific words used by participants, such as ‘recovery.’ For example, given that the word and broad construct ‘recovery’ was so prominent in initial coding, the analysis team agreed to further specify aspects of recovery in several codes (e.g., ‘12-step’ and ‘recovery factors'). This specificity fostered a coding process that generated codes closely mapped to participant meaning and ensured consistency among coders. The analysis team compared codes and came to a consensus where codes differed. Then, each transcription was reviewed again with this final coding list (horizontalization) which included a final list of 65 codes. The horizontalization process was utilized to give equal weight to all examined data. From this initial analysis, the analysis team organized statements from each transcript into meaning clusters. Next, these meaning clusters were developed into overall themes. These themes are explained in detail in the *Results* section*.*

All questionnaire responses were uploaded into a qualitative data analysis program (Dedoose, version 8.0.35) [37]. Upon completion of coding in Dedoose, responses were exported into Microsoft Excel (Version 16.43) and dummy coded as “1” or “0” to represent the presence or absence, respectively, of secondary and tertiary codes. Descriptive analyses were then conducted in RStudio (Version 4.0.2) [38] to analyze the frequency of codes. This approach of describing the frequency of codes allows one to see how common or rare a particular theme is and provides additional context to exploratory research on novel phenomenon (e.g., [39, 40]), but importantly, the frequency of a code is not meant to convey a proxy for statistical significance [35, 41, 43].

To ensure credibility and trustworthiness, one researcher acted as an internal auditor and bracketing strategies were used to externalize the personal beliefs and assumptions of the research team throughout analysis [42]. The analysis team used memos to monitor their reactions to the findings as data emerged. Regular debriefing sessions were used to review biases and discuss themes emerging in personal memos. Throughout the data analysis process, the analysis team considered if and/or how the emerging findings were informed by their initial beliefs and assumptions.

## Results

Students in the analytic sample (*N* = 92) identified as female (46.7%), male (50.0%), transgender (2.2%), and gender non-binary (1.1%) and had a mean age of 21.5 years^3^ (*SD* = 5.6). Among 73 students who reported their race, 64 identified as white (87.7%), four identified as multiracial, Hispanic (*n* = 3), and Asian (*n* = 1). In terms of mental health and substance use characteristics of this sample, 72% reported a history of mental health concerns and 25% reported a family history of addiction. The average number of days in recovery reported among 87 respondents was 437.1 (*SD* = 1079.4). Among 80 respondents, 33 reported their primary substance of choice as alcohol (41.3%), 18 students reported opiates (22.5%), 14 reported cannabis (17.5%), nine reported stimulants (11.3%), five students reported “other drugs” (6.3%), and one reported theirs as hallucinogens (1.3%).

Among 92 student responses, 95.7% of participants responded to all five prompts (one participant answered only two and three participants each answered four of the five total prompts). The average number of words per response across all five prompts was 24.6, with a range of 1–219 words across the 92 participants. Table 1 presents the average words per prompt, as well as the mode and range for each prompt.

**Table 1:** Descriptive statistics of responses by prompt

	Mean	Mode	Range	*n*
*What led you to seek recovery?*	19.0	8	1–11	92
*Describe success and challenges in your recovery journey*	30.8	17	1–218	91
*Describe your personal recovery program*	25.8	26	1–147	91
*What have been the most sustaining elements of your recovery?*	18.4	7	1–148	91
*What challenges will you face while trying to be successful in college and maintaining a healthy recovery program? What would be helpful to you in addressing these challenges?*	29.0	16	1–219	89

Mean, mode, and range of number of written words provided by the total sample (N = 92) per prompt

Two categories emerged from coding responses from 92 participants: (a) intrapersonal factors and (b) interpersonal/social factors. Each of the two categories included its own themes and subthemes. The following sections will describe these categories in detail.

Within these two categories, nine total themes emerged. First, within Intrapersonal Factors were five themes: recovery-specific challenges, self-care and coping, mental and behavioral health, life challenges, and personal motivations and attributes. Within Interpersonal Factors were the following four themes: 12-step recovery supports, external supports and community, college environment, and relationships with others. Each theme had several subthemes, which are outlined in Table 2 along with the percentage of participant responses endorsing each theme. Importantly, participants’ responses were often coded with multiple themes. Therefore, percentages may sum to a value greater than 100%.

**Table 2:** Categories, themes, and subthemes

Category: Intrapersonal factors			Category: Interpersonal/social factors	
Themes/Subthemes		*n* (%) participants	Themes/Subthemes	*n* (%) participants
Recovery-specific challenges Adopting “recovery first” mindset Ecological changes Resisting triggers Recovery resources Struggle aligning with recovery goals Slips/return to use		72 (78.3%)	12-step recovery supports Higher power “Life was unmanageable” Mantras Meetings Sponsorship Step work	76 (82.6%)
Self-care and coping skills Communication/reaching out Exercise/physical activity Maintain/create balance Meditation/mindfulness Prayer Sober activities Stress management Time management Weekend activities		62 (67.4%)	External supports and community Campus-based community Community service Encouragement from others General social support Recovery community (CRP)/ Sober network Sober housing	70 (76.1%)
Mental and behavioral health Addiction dependency symptoms Anxiety/depression Group and milieu therapy Other behavioral health challenges Substance use/mental health treatment Wanting to die		52 (56.5%)	College environment Academic challenges Academic successes Navigating social life in college Abstinence hostile environment	54 (58.7%)
Life challenges Feeling different Financial challenges Negative life events Sources of stigma		39 (42.4%)	Relationships with others Family dynamics (support and history of substance use) Helping others Re-establishing/repairing relationships Relational harm Romantic relationships	36 (39.1%)
Personal motivations and attributes Accountability Desire for change Future orientation (life goals, purpose, new mindset) Honesty Self-efficacy		38 (41.3%)		

Themes and subthemes identified through iterative coding process and percentage of sample (*N* = 92) who endorsed each theme

### Category: Intrapersonal factors

#### Themes and subthemes

##### Recovery-specific challenges

Participants discussed various challenges that were specific to the experience of being “in recovery” or seeking recovery from substance use (*n* = 72; 78.3%). Some discussed situations that impacted their recovery and others reported challenges that led them to initially seek and subsequently sustain recovery. They spoke about adapting to the motto of “recovery first” and having a “recovery mindset,” meaning holding recovery as their priority. Additional challenges specific to being in recovery included slips/returning to use, navigating triggers for reuse, ecological changes required to align with recovery (e.g., moving schools, towns), and resisting temptations to use/re-use. One quote from a female participant highlighted the subtheme “ecological changes:” *…at first I didn't realize I had to change everything, people, places, and things included.* Another female participant wrote about challenges they faced along their recovery journey, including cravings even after a period of sobriety: *7 months of continuous sobriety. Still find myself craving Percocet.*

##### Self-care and coping skills

This theme included participant-derived self-care strategies and coping skills, endorsed by two-thirds of the sample (*n* = 62; 67.4%) These included individual strategies and actions such as communication (e.g., reaching out to their supports), stress and time management, exercise, mindfulness/meditation, and prayer. Participants also identified sober activities, including ones they could engage in on the weekends. Finally, participants discussed an overarching goal of maintaining and achieving “balance” in their life using self-care practices and coping skills. For example, one female participant said: *Learning how to balance my recovery and schoolwork together so that I don't drift away from my primary purpose, which is staying sober.* Similarly, one female participant spoke about the challenges of “taking on too much” while in school and being cognizant of this in relation to their coping strategies. Another participant (male) highlighted the following coping strategies: *Exercise and eating healthy along with connecting with others in recovery and good friends.* A final quote highlights one female participant’s personal recovery program in which they identified a blend of self-care strategies and recovery supports: *I find it important to be* + *am currently involved in recovery-based group therapy. Self-care, honest* + *healthy relationships,* + *focus on school/work that I enjoy all help stabilize me.*

##### Mental and behavioral health

College students seeking recovery services also wrote about their experiences with other mental and behavioral health concerns, as well as treatment experiences (*n* = 52; 56.5%). In terms of specific mental health challenges, anxiety, depression, wanting to die (i.e., past or present suicidal thoughts/feelings), substance use dependency symptoms (e.g., cravings, physically could not stop using), and other behavioral health concerns (e.g., disordered eating, gambling) were commonly discussed. A male participant wrote about anxiety, highlighting the interconnected cycle between managing recovery and their mental health: *Anxiety issues will keep coming up, and returning to substances would make my anxiety worse because it would give me real things to be anxious about.* Others wrote about past experiences of mental health challenges and how these were connected to their substance use prior to being in recovery: *I had been struggling with depression for several years, and I was using alcohol to self-medicate. The alcohol abuse started to get out of control, so I reached out for help, and eventually started my recovery…*(female participant). These quotes highlight how participants bring to college with them a plethora of mental and behavioral health challenges, both past and present.

##### Life challenges

Another theme that emerged was “life challenges,” which captured broader struggles that many young adults face but that were particularly salient for this sample of students in recovery. This theme was reflected by the responses of 39 participants (42.4%) and included the following subthemes: sources of stigma, feeling different, financial struggles, and negative life events. One participant (female) shared the following quote: *I would say one of the biggest challenges was becoming "ready" and accepting the fact that I, as a young adult, living in a world where alcohol is constantly used and glamorized, am an alcoholic. It has been a difficult road accepting that I am "different" and cannot partake in regular activities that my peers can, and sometimes feel very left out and separate from everyone…* Another female participant wrote about how external life events, in addition to ecological changes (i.e., moving) created challenges for their recovery: *A lot of people I loved have died. Moving to [state] and feeling like a newcomer again was a huge challenge.* Additionally, many young adults and college students face financial difficulties, but for students in recovery, this life stressor can be an additional threat to their recovery: *I am financially insecure which gets very troublesome sometimes. I also lack a healthy support system at home* (female participant).

##### Personal motivations and attributes

The fifth intrapersonal theme identified consisted of participants’ personal motivations and characteristics that have impacted their recovery journey, endorsed by 38 participants (41.3%). This theme emerged from participants predominately noting their own reasons for seeking recovery and sharing which personal motivations and attributes helped them to sustain recovery and their goals. One male participant shared that they needed a change and decided to seek help for their substance use: *I wanted help because I got sick of the way I was living and feeling from drinking all the time.* Additionally, one female participant wrote about the most sustaining elements of their recovery: *Honestly, staying open, and constant willingness. Doing things I don't want to do, (in a good way). Pushing myself to be a better person and to love myself.*

### Category: Interpersonal/social factors

#### Themes and subthemes

##### Twelve-step recovery supports

The majority of participants (*n* = 76; 82.6%) discussed their participation in 12-step communities (e.g., Alcoholics Anonymous, Narcotics Anonymous) as a main resource in their recovery experience. The first step in the 12-step program consists of admitting that life has become unmanageable. Consistent with this, many participants wrote about this component as their cue to make changes in their substance use and therefore their recovery journey. One female participant stated: *To put it plainly, my life was unmanageable. Alcohol and drugs weren't making me feel better anymore, but I physically couldn't stop and had no idea what life was possible without them. I wanted to die but failed at that, so I tried the steps*. Others discussed how they apply what they have learned in 12-step in their day to day lives, including the principle of “honesty.” For example, one female participant shared: *The most sustaining elements of my recovery have been participating in a 12-step program and getting honest. There is no way I could have stayed in recovery this long had I not started to work a program and practice rigorous honesty.*

##### External supports and community

Most participants wrote about the importance of their communities and social support, including supports from their recovery community (including sober housing) but also from the broader community (*n* = 70; 76.1%). Participants wrote about their desire for encouragement from others, a campus community, and a general community beyond campus. For instance, one female individual wrote about engaging in community service and having a community outside of college: *Honestly it was [cycling class], the community there made me feel at home, even though it was not recovery based.* Many wrote about the need for general social support while in college, and how this was a helpful element to their recovery journey. For example, one male participant said: *Making friends that enjoy similar sober activities. I will connect with other participants in recovery and participate in clubs. Also, I'll attend local meetings.* In another instance, a male participant wrote: *The most sustaining elements of my recovery have been…working in a business environment where I have been very successful, becoming reconnected with my community of faith, and getting back to my community service roots.*

##### College environment

Another theme that emerged was related to the college environment itself, which often served as a motivator for seeking recovery services. Participants whose responses reflected this theme (*n* = 54; 58.7%) specifically identified the following subthemes: navigating social life, school breaks, and academic challenges and successes. One male participant wrote: *I am concerned about having to live around college students who frequent parties. I never want an incident like the one at [college] to happen again, and I am confident it won't.* Similarly, one male participant wrote about the common experience of feeling stress due to schoolwork, and the unique challenges of being a person in recovery in college: *Anxiety from school work will be a challenge, as will the social drinking settings prevalent on every college campus…* Similarly, another participant (female) highlighted their understanding of how the college environment will be challenging to navigate as they aim to find social outlets: *Many students my age use alcohol or drugs so I need to find substance-free ways to entertain myself. Sometimes I find it difficult to maintain focus in school because I lack a social outlet. I am seeking a healthy social network and I believe the CRC [collegiate recovery community] offers that.* Participants further noted the value of accessing recovery resources on campus, including sober college housing, in maintaining their recovery.

##### Relationships with others

The final theme that emerged related to relationships with others, reflected in responses by 36 participants (39.1%). This theme encompassed positive relationships, as well as interpersonal factors specific to recovery, such as experiences of causing relational harm due to substance use and/or repairing relationships with others. For example, one female participant wrote about the ways that their relationships have changed over the course of their recovery journey: *My challenges in recovery have been building my family's trust back and when I relapsed in [date] and had to go away to inpatient treatment*. Other participants wrote about their relationships as motivators and ways they have sustained their recovery in college. For instance, one male participant stated: *Studying keeps me entertained and happy. Friends and a romantic relationship are also key too.* Others identified family relationships as presenting barriers to their recovery, such as one male participant: *Challenges [to recovery journey]: living at home in unhealthy family dynamic.*

## Discussion

The primary aim of this study was to explore students’ experiences of seeking recovery in the college environment. We used a phenomenological approach to conduct a thematic analysis of written responses from student applications to a CRP. Themes and subthemes that emerged were coded and organized within two categories: intrapersonal factors and interpersonal/social factors. These categories are consistent with the recovery capital literature [18, 44], which highlights the various factors that can promote or hinder recovery from an ecological perspective. Themes that emerged from the data included: recovery-specific challenges, self-care and coping, mental and behavioral health, life challenges, and personal motivations and attributes, 12-step recovery supports, external supports and community, college environment, and relationships with others. Within these themes, students described the barriers and facilitators for their recovery, including drawing on personal attributes, coping supports, navigating the challenges and supports of the college environment, and addressing mental health and life challenges.

These findings broadly align with those of previous studies applying qualitative methodologies to the study of recovery in the context of CRPs [9, 21, 22]. For instance, Iarussi and colleagues [22] identified similar themes related to stigma, the impact of recovery on academics, changes in relationships, and recovery-based services and resources. Findings related to navigating college environments, feelings of isolation, experiences of stigma, and the roles of mutual support in sustaining recovery in college have also been identified in prior studies [9, 21]. Finally, our findings support prior evidence highlighting the unique challenges students in recovery may experience in prioritizing academic expectations with vocational goals and recovery needs while in college [9, 22].

Our findings also support and extend previous findings that students in recovery experience unique challenges in college, which can impact their academic, health, and recovery outcomes ([9, 19, 48]), as well as several key facilitators to recovery, which may be further linked to improved academic and mental health-related outcomes ([44, 48, 49]). Identified barriers include navigating the college environment (e.g., high endorsement of substance use, academic expectations); social isolation and stigma; challenging social relationships or family dynamics; life challenges; and navigating co-occurring mental illness. On the other hand, key facilitators include access to recovery resources (e.g. CRPs, housing, sober activities); finding and maintaining supportive peer networks; access to mental health services (e.g., university counseling services); and building and maintaining personal coping strategies (e.g., mindfulness, stress management, and time management). Participants also cited the value of fostering a personal sense of self-efficacy or recovery orientation while in college. They noted that specific personal motivations or mindsets may buffer the challenges to sustainable recovery in the college environment. These may inspire the use of support resources, such as reaching out to social networks or engaging with CRPs.

This study contributes to the qualitative literature base of college student recovery experiences by (a) assessing written responses to questionnaires in a relatively large sample of college students seeking recovery and admission into a CRP, and (b) exploring the utility and applicability of open-ended written data gathered through the application or intake process of CRPs to better understand student experiences. This approach to qualitative analysis offers insight into students’ motivations for accessing services and goals for engagement with CRPs, which can inform how CRPs conduct outreach and develop services to best address student needs. By assessing written responses to questionnaires through structural and textual cluster analysis [35], the current study makes important contributions to the literature by analyzing data from a relatively large sample, thereby bolstering and expanding upon the existing evidence base. Open-ended written data gathered through the application or intake process may offer insight into motivations for accessing services and goals for engagement with CRPs. Given that CRPs collect a variety of student data [12], future scholarship may benefit from increased gathering of program-embedded data (e.g., intakes, tracking forms, goal setting, or advising meetings) alongside academic outcomes, counseling services, or health-related data to better understand student experiences over time, as well as to better understand related academic and health outcomes.

College students experience barriers and facilitators specific to college life that can impact their academic and recovery outcomes [19, 45]. College environments themselves present a challenge as substance use is often endorsed and centered in social activities. Students reported feeling socially isolated and stigmatized in this environment. Academically, students reported struggling to balance expectations with prioritizing needs related to their health and recovery. Given increases in help-seeking behavior among college students over the past decade [46], CRPs and other college-based student supports are in a unique position to provide direct intervention. In addition, given what students reported about substance use in the college environment and stigma, CRPs should engage in community outreach and prevention efforts to address substance use among college students and reduce stigma around college students’ substance-related choices and experiences.

Recognizing that college students may enter college connected with pre-existing recovery resources, CRPs should strive to connect with off-campus or outside resources and community agencies to ensure smooth transitions between services. Given that a primary concern voiced by students in recovery includes the need to balance academic, vocational, and recovery priorities, support in CRPs may extend beyond more traditional recovery support services by including academic skill building such as time management, study strategies, and test-taking support.

## Limitations

It is important to contextualize the findings of this study considering several limitations. First, this study is based on a convenience sample, limiting the generalizability of findings. Of the 13 CRPs contacted as part of the original parent study, only one CRP collected qualitative questionnaire data from students who were completing applications. This CRP was an abstinence-based program that required active participation and offered multiple weekly activities at a university with sober living options. As a result, data analyzed in this study may not be representative of CRPs at other colleges or the students who access their services. Additionally, these data were collected between 2011 and 2018, which may not accurately reflect the current trends and/or the state of CRPs and experiences of college students seeking recovery services and resources. Although students’ primary substance of choice, mental health and family histories, and time in recovery were included as descriptors in the current study, we could not examine how these or other salient factors may directly influence the resources and types of services that students seek out in their recovery journey (e.g., [47]). Finally, we relied on this secondary data to minimize data collection burden,qualitative data were self-reported and only a subset of the entire applicant sample (56%) contributed responses. It was not triangulated with other data collection strategies, which may result in sources of bias such as perceived desirability.

## Conclusions

The aims of the present study were to explore the experience of recovery from students’ responses to questions about recovery needs, challenges, and experiences while also attending college. CRPs may offer crucial support to students in recovery, who must meet academic demands while maintaining their personal well-being and recovery goals in the college environment. Given these varying and often competing demands, CRPs may extend beyond more traditional recovery support services by addressing academic and vocational needs, in addition to offering social support resources for mental and behavioral health. Findings from the present study suggested that students seeking recovery supports from CRPs feel socially isolated and stigmatized, are attempting to find balance in their academic and personal lives, and are utilizing self-care and coping strategies to support their mental and behavioral health, and their recovery goals. In addition to offering tangible academic and recovery resources, CRPs may specifically support college students by offering recovery-oriented social spaces and supporting students in developing strategies to maintain recovery goals specific to college socializing and belonging. CRPs may also be the first point of contact for students seeking specific supports to navigate the academic and social environment of college, as well as offering continued and/or new supports as students navigate their mental health needs in college. Given the increased barriers for students in recovery, CRPs should work closely with their campus wellness and counseling centers to ensure CRP students have direct access to mental health referrals. CRPs could also find trusted behavioral health referrals in the surrounding community that understand the unique experiences of students in recovery. Further, CRPs are well-positioned to address community-level change, for example by engaging in outreach or prevention efforts to reduce substance use and recovery-related stigma.

## Data Availability

The datasets analyzed during the current study are not publicly available due to restrictions established in the data sharing agreements with participating sites and the potentially identifiable nature of the data.

## Abbreviations

SUDs: Substance use disorders
CRPs: Collegiate recovery programs
CRCs: Collegiate recovery communities

## Footnotes

1 Students who responded to the qualitative prompts (*n* = 92) versus those who did not (*n* = 71) were slightly younger, *t*(155.6) = 2.52, *p* = .012, and reported significantly less time in recovery, *t*(149.9) = 3.05, *p* = .002. There were no statistically significant differences in responders to the qualitative questions (versus non-responders) in terms of race, sex, mental health history, or family history of addiction.

2 The fifth prompt contained a two-part question.

3 The mean age of the sample is based on responses from 91 students (*n* missing = 1).
